# The Revised Self-Monitoring Scale detects early impairment of social cognition in genetic frontotemporal dementia within the GENFI cohort

**DOI:** 10.1186/s13195-021-00865-w

**Published:** 2021-07-12

**Authors:** Hannah D. Franklin, Lucy L. Russell, Georgia Peakman, Caroline V. Greaves, Martina Bocchetta, Jennifer Nicholas, Jackie Poos, Rhian S. Convery, David M. Cash, John van Swieten, Lize Jiskoot, Fermin Moreno, Raquel Sanchez-Valle, Barbara Borroni, Robert Laforce, Mario Masellis, Maria Carmela Tartaglia, Caroline Graff, Daniela Galimberti, James B. Rowe, Elizabeth Finger, Matthis Synofzik, Rik Vandenberghe, Alexandre de Mendonça, Fabrizio Tagliavini, Isabel Santana, Simon Ducharme, Chris Butler, Alex Gerhard, Johannes Levin, Adrian Danek, Markus Otto, Sandro Sorbi, Isabelle Le Ber, Florence Pasquier, Jonathan D. Rohrer, on behalf of the Genetic FTD Initiative, GENFI, Sónia Afonso, Maria Rosario Almeida, Sarah Anderl-Straub, Christin Andersson, Anna Antonell, Silvana Archetti, Andrea Arighi, Mircea Balasa, Myriam Barandiaran, Nuria Bargalló, Robart Bartha, Benjamin Bender, Alberto Benussi, Maxime Bertoux, Anne Bertrand, Valentina Bessi, Sandra Black, Sergi Borrego-Ecija, Jose Bras, Alexis Brice, Rose Bruffaerts, Agnès Camuzat, Marta Cañada, Valentina Cantoni, Paola Caroppo, Miguel Castelo-Branco, Olivier Colliot, Thomas Cope, Vincent Deramecourt, María de Arriba, Giuseppe Di Fede, Alina Díez, Diana Duro, Chiara Fenoglio, Camilla Ferrari, Catarina B. Ferreira, Nick Fox, Morris Freedman, Giorgio Fumagalli, Aurélie Funkiewiez, Alazne Gabilondo, Roberto Gasparotti, Serge Gauthier, Stefano Gazzina, Giorgio Giaccone, Ana Gorostidi, Caroline Greaves, Rita Guerreiro, Carolin Heller, Tobias Hoegen, Begoña Indakoetxea, Vesna Jelic, Hans-Otto Karnath, Ron Keren, Gregory Kuchcinski, Tobias Langheinrich, Thibaud Lebouvier, Maria João Leitão, Albert Lladó, Gemma Lombardi, Sandra Loosli, Carolina Maruta, Simon Mead, Lieke Meeter, Gabriel Miltenberger, Rick van Minkelen, Sara Mitchell, Katrina Moore, Benedetta Nacmias, Annabel Nelson, Linn Öijerstedt, Jaume Olives, Sebastien Ourselin, Alessandro Padovani, Jessica Panman, Janne M. Papma, Yolande Pijnenburg, Cristina Polito, Enrico Premi, Sara Prioni, Catharina Prix, Rosa Rademakers, Veronica Redaelli, Daisy Rinaldi, Tim Rittman, Ekaterina Rogaeva, Adeline Rollin, Pedro Rosa-Neto, Giacomina Rossi, Martin Rossor, Beatriz Santiago, Dario Saracino, Sabrina Sayah, Elio Scarpini, Sonja Schönecker, Harro Seelaar, Elisa Semler, Rachelle Shafei, Christen Shoesmith, Imogen Swift, Miguel Tábuas-Pereira, Mikel Tainta, Ricardo Taipa, David Tang-Wai, David L. Thomas, Paul Thompson, Hakan Thonberg, Carolyn Timberlake, Pietro Tiraboschi, Emily Todd, Philip Van Damme, Mathieu Vandenbulcke, Michele Veldsman, Ana Verdelho, Jorge Villanua, Jason Warren, Carlo Wilke, Ione Woollacott, Elisabeth Wlasich, Henrik Zetterberg, Miren Zulaica

**Affiliations:** 1 Dementia Research Centre, Department of Neurodegenerative Disease, UCL Queen Square Institute of Neurology, Queen Square, London, WC1N 3BG UK; 2 Department of Medical Statistics, London School of Hygiene and Tropical Medicine, London, UK; 3 Department of Neurology, Erasmus Medical Centre, Rotterdam, Netherlands; 4 Centre for Medical Image Computing, Department of Medical Physics and Biomedical Engineering, University College London, London, United Kingdom; 5 Cognitive Disorders Unit, Department of Neurology, Donostia University Hospital, San Sebastian, Gipuzkoa Spain; 6 Neuroscience Area, Biodonostia Health Research Institute, San Sebastian, Gipuzkoa Spain; 7 Alzheimer’s disease and Other Cognitive Disorders Unit, Neurology Service, Hospital Clínic, Institut d’Investigacións Biomèdiques August Pi I Sunyer, University of Barcelona, Barcelona, Spain; 8 Neurology Unit, Department of Clinical and Experimental Sciences, University of Brescia, Brescia, Italy; 9 Clinique Interdisciplinaire de Mémoire, Département des Sciences Neurologiques, CHU de Québec, and Faculté de Médecine, Université Laval, Québec, QC Canada; 10 Sunnybrook Health Sciences Centre, Sunnybrook Research Institute, University of Toronto, Toronto, Canada; 11 Tanz Centre for Research in Neurodegenerative Diseases, University of Toronto, Toronto, Canada; 12 Center for Alzheimer Research, Division of Neurogeriatrics, Department of Neurobiology, Care Sciences and Society, Bioclinicum, Karolinska Institutet, Solna, Sweden; 13 Unit for Hereditary Dementias, Theme Aging, Karolinska University Hospital, Solna, Sweden; 14 Fondazione Ca’ Granda, IRCCS Ospedale Policlinico, Milan, Italy; 15 University of Milan, Centro Dino Ferrari, Milan, Italy; 16 Department of Clinical Neurosciences, University of Cambridge, Cambridge, UK; 17 Department of Clinical Neurological Sciences, University of Western Ontario, London, Ontario Canada; 18 Department of Neurodegenerative Diseases, Hertie-Institute for Clinical Brain Research and Center of Neurology, University of Tübingen, Tübingen, Germany; 19 Center for Neurodegenerative Diseases (DZNE), Tübingen, Germany; 20 Laboratory for Cognitive Neurology, Department of Neurosciences, KU Leuven, Leuven, Belgium; 21 Neurology Service, University Hospitals Leuven, Leuven, Belgium; 22 Leuven Brain Institute, KU Leuven, Leuven, Belgium; 23 Laboratory of Neurosciences, Institute of Molecular Medicine, Faculty of Medicine, University of Lisbon, Lisbon, Portugal; 24 Fondazione IRCCS Istituto Neurologico Carlo Besta, Milano, Italy; 25 University Hospital of Coimbra (HUC), Neurology Service, Faculty of Medicine, University of Coimbra, Coimbra, Portugal; 26 Center for Neuroscience and Cell Biology, Faculty of Medicine, University of Coimbra, Coimbra, Portugal; 27 Department of Psychiatry, McGill University Health Centre, McGill University, Montreal, Québec Canada; 28 McConnell Brain Imaging Centre, Montreal Neurological Institute, McGill University, Montreal, Québec Canada; 29 Nuffield Department of Clinical Neurosciences, Medical Sciences Division, University of Oxford, Oxford, UK; 30 Division of Neuroscience and Experimental Psychology, Wolfson Molecular Imaging Centre, University of Manchester, Manchester, UK; 31 Departments of Geriatric Medicine and Nuclear Medicine, University of Duisburg-Essen, Duisburg, Germany; 32 Department of Neurology, Ludwig-Maximilians Universität München, Munich, Germany; 33 German Center for Neurodegenerative Diseases (DZNE), Munich, Germany; 34 Munich Cluster of Systems Neurology (SyNergy), Munich, Germany; 35 Department of Neurology, University of Ulm, Ulm, Germany; 36 Department of Neuroscience, Psychology, Drug Research and Child Health, University of Florence, Florence, Italy; 37 IRCCS Don Gnocchi, Firenze, Italy; 38 Sorbonne Université, Paris Brain Institute – Institut du Cerveau – ICM, Inserm U1127, CNRS UMR 7225, AP-HP - Hôpital Pitié-Salpêtrière, Paris, France; 39 Centre de référence des démences rares ou précoces, IM2A, Département de Neurologie, AP-HP - Hôpital Pitié-Salpêtrière, Paris, France; 40 Département de Neurologie, AP-HP - Hôpital Pitié-Salpêtrière, Paris, France; 41 Univ Lille, Lille, France; 42 Inserm 1172, Lille, France; 43 CHU, CNR-MAJ, Labex Distalz, LiCEND, Lille, France

**Keywords:** Frontotemporal dementia, Familial, *C9orf72*, *GRN*, *MAPT*, RSMS, CDR® plus NACC FTLD, VBM

## Abstract

**Background:**

Although social cognitive dysfunction is a major feature of frontotemporal dementia (FTD), it has been poorly studied in familial forms. A key goal of studies is to detect early cognitive impairment using validated measures in large patient cohorts.

**Methods:**

We used the Revised Self-Monitoring Scale (RSMS) as a measure of socioemotional sensitivity in 730 participants from the genetic FTD initiative (GENFI) observational study: 269 mutation-negative healthy controls, 193 *C9orf72* expansion carriers, 193 *GRN* mutation carriers and 75 *MAPT* mutation carriers. All participants underwent the standardised GENFI clinical assessment including the ‘CDR® plus NACC FTLD’ scale and RSMS. The RSMS total score and its two subscores, socioemotional expressiveness (EX score) and modification of self-presentation (SP score) were measured. Volumetric T1-weighted magnetic resonance imaging was available from 377 mutation carriers for voxel-based morphometry (VBM) analysis.

**Results:**

The RSMS was decreased in symptomatic mutation carriers in all genetic groups but at a prodromal stage only in the *C9orf72* (for the total score and both subscores) and *GRN* (for the modification of self-presentation subscore) groups. RSMS score correlated with disease severity in all groups. The VBM analysis implicated an overlapping network of regions including the orbitofrontal cortex, insula, temporal pole, medial temporal lobe and striatum.

**Conclusions:**

The RSMS indexes socioemotional impairment at an early stage of genetic FTD and may be a suitable outcome measure in forthcoming trials.

**Supplementary Information:**

The online version contains supplementary material available at 10.1186/s13195-021-00865-w.

## Background

Frontotemporal dementia (FTD) is a complex and heterogeneous neurodegenerative disease, manifesting itself as a diverse spectrum of clinical syndromes. However, despite differences in presentation, many people with FTD develop impaired social cognition [1], a set of psychological processes which includes the ability to evaluate social and emotional cues from others and then select an appropriate behavioural response, a phenomenon often referred to as ‘socioemotional sensitivity’ or ‘self-monitoring’. In both healthy and clinical populations, the Revised Self-Monitoring Scale (RSMS) [2] has often been used to study socioemotional sensitivity and responsiveness as well as the neural networks that underlie them [3, 4].

Unlike many neurodegenerative diseases, FTD is highly heritable with approximately a third of patients having a causative autosomal dominant genetic mutation [5]. Mutations are most commonly found in one of three genes, chromosome 9 open reading frame 72 (*C9orf72*), progranulin (*GRN*) and microtubule-associated protein tau (*MAPT*) [6], with the most common clinical presentation being behavioural variant FTD (bvFTD) [7]. However, whilst social cognitive dysfunction has been studied extensively in sporadic FTD, few investigations have looked at genetic cohorts exclusively.

The Genetic FTD Initiative (GENFI) is a multicentre natural history study aimed at investigating early biomarkers in a large genetic FTD cohort, including measures of cognition [5]. This study sought to assess whether the RSMS could detect early changes in social cognition and what the underlying neural correlates of the RSMS were in people with mutations in *C9orf72*, *GRN* and *MAPT*.

## Methods

### Participants

Participants were recruited from the fifth data freeze of GENFI, incorporating data from 24 sites. Of the 849 participants enrolled in the second phase of the study, cross-sectional data on the RSMS was available from 730 participants, consisting of 269 healthy controls (family members who tested negative for the mutation carried within the family), 193 *C9orf72* expansion carriers, 193 *GRN* mutation carriers and 75 *MAPT* mutation carriers (Table 1). All participants provided written informed consent.

**Table 1:** Demographics and the RSMS total, EX and SP scores for each genetic group, split by global CDR® plus NACC FTLD score (0, 0.5, 1+). N represents number of participants, mean (standard deviation) shown for age, education and cognitive test scores. In the *symptomatic* (1+) groups, MMSE scores were significantly lower in *GRN* mutation carriers than in the *C9orf72* expansion carrier group but no other differences were seen, whilst no differences were seen in the CDR® plus NACC FTLD-SB

		N	Sex	Age (years)	Education (years)	MMSE (/30)	CDR plus NACC FTLD-SB	RSMS total (/65)	RSMS EX (/30)	RSMS SP (/35)
			% male	Mean (SD)	Mean (SD)	Mean (SD)	Mean (SD)	Mean (SD)	Mean (SD)	Mean (SD)
**Controls**		**269**	42	46.2 (13.0)	14.4 (3.4)	29.3 (1.1)	0.2 (0.4)	47.8 (8.4)	23.3 (4.2)	24.5 (5.3)
***C9orf72***	**0**	**93**	41	43.9 (11.6)	14.3 (3.0)	29.1 (1.2)	0.0 (0.0)	47.1 (10.5)	22.8 (5.4)	24.3 (6.0)
	**0.5**	**34**	44	49.7 (11.2)	14.0 (2.6)	28.4 (2.2)	1.1 (0.7)	41.9 (11.4)	19.8 (6.2)	22.1 (6.3)
	**1+**	**66**	65	62.7 (9.5)	13.0 (3.8)	23.3 (6.8)	11.1 (5.6)	23.5 (12.3)	9.6 (7.0)	14.0 (6.6)
***GRN***	**0**	**122**	34	45.6 (12.2)	14.7 (3.5)	29.5 (0.8)	0.0 (0.0)	47.9 (8.9)	23.6 (4.0)	24.3 (5.9)
	**0.5**	**24**	46	51.3 (13.8)	14.0 (4.3)	28.6 (2.3)	0.9 (0.8)	43.8 (10.7)	21.6 (6.3)	22.2 (5.6)
	**1+**	**47**	47	63.0 (7.4)	11.7 (3.4)	20.1 (7.7)	9.8 (6.2)	28.6 (12.1)	12.9 (6.7)	15.6 (6.1)
***MAPT***	**0**	**41**	41	38.3 (11.0)	14.3 (3.3)	29.5 (0.8)	0.0 (0.0)	50.7 (9.7)	24.0 (4.5)	26.7 (6.0)
	**0.5**	**13**	31	46.4 (12.8)	13.6 (2.5)	28.1 (2.3)	1.1 (0.8)	50.1 (14.2)	23.8 (7.5)	26.3 (7.1)
	**1+**	**21**	57	58.9 (9.4)	13.6 (4.0)	21.9 (8.1)	10.3 (6.0)	22.8 (18.9)	9.4 (9.5)	13.4 (9.8)

### Assessments

All participants were given the standardised GENFI clinical assessment battery including a medical history, physical examination, the Mini-Mental State Examination, and the CDR® Dementia Staging Instrument with the National Alzheimer Coordinating Centre Frontotemporal Lobar Degeneration component (CDR® plus NACC FTLD) (Table 1). The CDR® plus NACC FTLD is a clinical measure of disease severity in FTD, consisting of a core six cognitive/functional domains with a further 2 domains addressing behaviour and language [8]. Each domain is rated on a five-point scale ranging from 0 (normal), 0.5 (questionably or minimally impaired), 1 (mildly but definitely impaired), 2 (moderately impaired), to 3 (severely impaired). The sum of ratings across all eight domains is used to generate the CDR® plus NACC FTLD *sum of boxes* (CDR® plus NACC FTLD-SB) (Table 1). A second measure, a *global* CDR® plus NACC FTLD score can also be generated, using a specific algorithm [9]. We used this global score to classify each of the genetic groups cross-sectionally into those who scored 0 (i.e. were asymptomatic), 0.5 (possibly or mildly symptomatic i.e. prodromal), and 1 or more (fully symptomatic mutation carriers). A neuropsychological assessment was also performed including the Trail Making Test Parts A and B, the WAIS-R Digit Symbol test, the D-KEFS Color-Word Interference Test Ink Naming, category fluency (animals), the Faux Pas recognition test, and the Facial Emotion Recognition Test.

### Demographics

Demographics are shown in Table 1. There was a significant difference in sex between these groups: symptomatic *C9orf72* carriers had a significantly higher percentage of males than in the mildly symptomatic and asymptomatic *C9orf72* carrier groups and in the controls (X^2^(1) = 4.08, *p* = 0.044, X^2^(1) = 9.12, *p* = 0.003 and X^2^(1) = 11.79, *p* = 0.001, respectively). There was also a significant difference in age between groups (F(9,720)) = 27.5, *p* < 0.001): asymptomatic *MAPT* mutation carriers were significantly younger and mildly symptomatic *GRN* mutation carriers were significantly older than controls (*p* < 0.001 and *p* = 0.043 respectively). All symptomatic mutation carriers were significantly older than controls (*p* < 0.001). Analysis of differences in years spent in education (F(9,720)) = 4.09, *p* < 0.001) showed that symptomatic *C9orf72* and *GRN* mutation carriers spent significantly fewer years when compared to controls (*p* = 0.003 and *p* < 0.001, respectively). All analyses were therefore adjusted for sex, age and education.

### Revised Self-Monitoring Scale (RSMS)

The RSMS is a widely used questionnaire made up of 13 items designed to measure an individual’s awareness of social behaviour and sensitivity to subtle emotional expressions during face-to-face interaction [10]. Items include ‘In conversations, the subject is sensitive to even the slightest change in the facial expression of the person he/she is conversing with’ and ‘If someone is lying to the subject, he/she usually knows it at once from that person’s manner or expression’. Each item is rated by a participant’s informant on a 6-point scale, ranging from ‘certainly, always false’ (0 points) to ‘certainly, always true’ (6 points). As well as a total score, two subscores of the RSMS can also be calculated: socioemotional expressiveness i.e. the ability to understand subtle social cues in others (EX score, out of 30), and modification of self-presentation i.e. the ability to change one’s behaviour when it is not appropriate for the current social situation (SP score, out of 35).

### Statistical analysis

Statistical analyses were performed using StataCorp. 2019. Stata Statistical Software: Release 16. College Station, TX: StataCorp LLC. In the healthy control group, Spearman rank correlations were performed to assess the relationship between the RSMS total score, age, sex and education. Cross-sectional RSMS total, EX and SP scores were compared between groups (healthy controls, and 0, 0.5 and 1+ in each genetic group) using a linear regression model adjusting for age, sex and education, with 95% bias-corrected bootstrapped confidence intervals with 1000 repetitions (to correct for non-normally distributed data). Spearman rank correlations were performed in each genetic group to investigate the association between RSMS total score and disease severity (as measured by CDR® plus NACC FTLD-SB). Finally, non-parametric partial correlations adjusting for age, sex, education and disease severity were also performed to assess the relationship between RSMS total score and cognition.

### Image acquisition and processing

Participants underwent volumetric T1-weighted magnetic resonance imaging according to the harmonized GENFI protocol on a 3T scanner. All images underwent quality control and any scans with movement or artefacts were eliminated from analysis. In addition, any scans displaying moderate to severe vascular disease or any lesion presentation were also excluded. 377 scans were included in the analysis: 151 *C9orf72* expansion carriers, 162 *GRN* mutation carriers and 64 *MAPT* mutation carriers. Voxel-based morphometry (VBM) was subsequently performed using Statistical Parametric Mapping (SPM) 12(www.fil.ion.ucl.ac.uk/spm), running under Matlab R2014a (Mathworks, USA). T1-weighted images were normalised and segmented into grey matter (GM), white matter (WM) and cerebrospinal fluid (CSF) probability maps using standard procedures and a fast-diffeomorphic image registration algorithm (DARTEL) [11]. Prior to analysis, GM segmentations were then transformed into Montreal Neurological Institute (MNI) space, modulated and smoothed using a Gaussian kernel with 6-mm fill-width at half maximum, before applying a mask image as reported in Ridgway et al. 2009 [12]. In order to investigate the neural correlates of socioemotional sensitivity in each genetic group, multiple regression models were performed to explore the relationship of RSMS total score and GM density in mutation carriers in each genetic group. Age, sex, scanner type and total intracranial volume (TIV, calculated using SPM [13]) were included as nuisance covariates. The Family-Wise Error (FWE) correction for multiple comparisons was set at 0.05. However, if no findings were observed at that strict level of correction, results were reviewed at an uncorrected p value of 0.001.

## Results

### Healthy control performance on the RSMS

Mean (standard deviation) RSMS total score was 47.8 (8.4) in controls (Tables S1 and S2). Overall, there was no significant difference between performance in females (n = 157: 48.5 (8.0)) and males (n = 112: 46.8 (9.0) (*p* = 0.21). No significant correlations between RSMS total score and age (rho = 0.01, *p* = 0.87) or education (rho = 0.12, *p* = 0.06) were observed.

### Cross-sectional analysis of mutation carriers

**Fig. 1:**
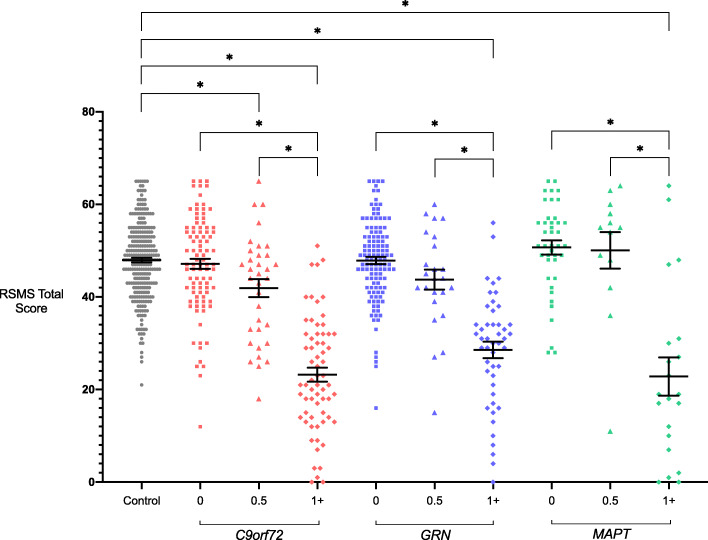
RSMS total scores in each genetic group, stratified by global CDR® plus NACC FTLD scores. Bars represent the mean score and standard error of the mean in each group. Significant differences from controls and within each genetic group are starred. Differences between different genetic groups are not shown

Mean RSMS total scores in all symptomatic (CDR 1+) mutation carriers were significantly lower than in healthy controls (Tables 1 and 2, Fig. 1): *C9orf72* 23.5 (12.3), *GRN* 28.6 (12.1) and *MAPT* 22.8 (18.9). In the CDR 0.5 groups, the *C9orf72* group also scored significantly lower than controls with a trend for a lower score in the *GRN* group and no difference in the *MAPT* group: *C9orf72* 41.9 (11.4), *GRN* 43.8 (12.1) and *MAPT* 50.1 (14.2). No significant differences were observed between the asymptomatic (CDR 0) mutation carrier groups and controls.

**Table 2:** Adjusted mean differences in RSMS total scores between the genetic groups stratified by global CDR® plus NACC FTLD scores, with 95% bias-corrected confidence intervals. Significant values are shown in bold

		***C9ORF72***						***GRN***						***MAPT***					
		0		0.5		1+		0		0.5		1+		0		0.5		1+	
**Controls**		−0.91		−**5.34**		−**22.39**		−0.35		−4.05		−**17.41**		1.99		2.07		−**24.28**	
		−3.41	1.60	−**9.36**	**-1.32**	−**25.72**	−**19.05**	−2.17	1.47	−8.53	0.42	−**20.94**	−**13.88**	−1.21	5.19	−5.72	9.86	−**32.48**	−**16.08**
***C9ORF72***	**0**			−4.43		−**21.48**		0.55		−3.15		−**16.50**		2.90		2.97		−**23.37**	
				−9.10	0.24	−**25.68**	−**17.29**	−2.29	3.39	−8.19	1.90	−**20.84**	−**12.17**	−0.91	6.71	−5.02	10.97	−**31.89**	−**14.86**
	**0.5**					−**17.05**		**4.99**		1.29		−**12.07**		**7.33**		7.41		−**18.94**	
						−**21.93**	−**12.16**	**0.61**	**9.36**	−4.54	7.11	−**17.17**	−**6.98**	**2.19**	**12.48**	−1.18	15.99	−**27.88**	−**10.01**
	**1+**							**22.04**		**18.34**		**4.98**		**24.38**		**24.45**		−1.89	
								**18.41**	**25.66**	**12.93**	**23.74**	**0.40**	**9.55**	**19.79**	**28.97**	**16.28**	**32.63**	−10.40	6.61
***GRN***	**0**									−3.70		−**17.06**		2.35		2.42		−**23.93**	
										−8.60	1.20	−**20.77**	−**13.35**	−1.31	6.00	−5.61	10.44	−**32.26**	−**15.60**
	**0.5**											−**13.36**		**6.05**		6.12		−**20.23**	
												−**18.76**	−**7.96**	**0.72**	**11.38**	−2.59	14.83	−**29.17**	−**11.29**
	**1+**													**19.41**		**19.48**		−6.87	
														**14.54**	**24.27**	**11.10**	**27.85**	−15.59	1.85
***MAPT***	**0**															0.07		−**26.27**	
																−7.46	7.60	−**35.02**	−**17.53**
	**0.5**																	−**26.35**	
																		−**36.96**	−**15.74**
	**1+**

Within each genetic group, there was a significantly lower RSMS total score in the symptomatic group compared with the CDR 0.5 and CDR 0 groups (Tables 1 and 2, Fig. 1).

**Fig. 2:**
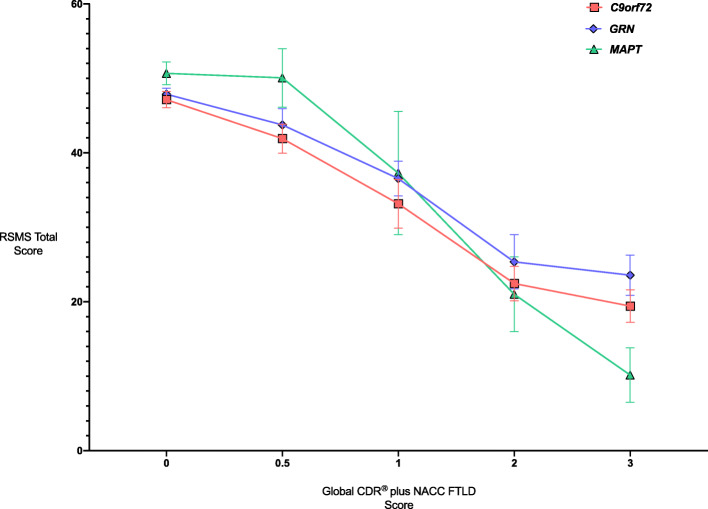
Mean RSMS total scores in each genetic group by individual global CDR® plus NACC FTLD score. Error bars represent standard error of the mean

Stratifying by individual global CDR® plus NACC FTLD score (0, 0.5, 1, 2 and 3), all genetic groups show decreasing RSMS total score with increasing CDR (Fig. 2).

RSMS EX and SP scores followed a similar pattern as for RSMS total performance (Table 1, Tables S3 and S4, Figures S1 and S2): the mean scores in all symptomatic (CDR 1+) mutation carriers and the *C9orf72* CDR 0.5 group were significantly lower than in healthy controls for both EX and SP scores. However, additionally, the *GRN* CDR 0.5 group had significantly lower mean SP score than controls. Within each genetic group, there was a significantly lower RSMS EX and SP score in the symptomatic groups compared with the CDR 0.5 and CDR 0 groups, with EX score also lower in the *C9orf72* CDR 0.5 group compared with the CDR 0 group (Tables S3 and S4, Figures S1 and S2).

### Relationship between RSMS and CDR® plus NACC FTLD-SB

A strong negative correlation between RSMS total score and CDR® plus NACC FTLD-SB scores was observed for all genetic groups (Figure S3)*: C9orf72* (r = −0.67, *p* < 0.001), *GRN* (r = −0.59, *p* < 0.001) and *MAPT* (r = −0.53, *p* < 0.001).

### Relationship between RSMS and cognition

A weak positive correlation was found between RSMS total score and one test of social cognition, the Facial Emotion Recognition test, in the *C9orf72* group only (r = 0.18, *p* = 0.018; Table S5). However, no significant correlations were found on other tests of cognition except for category fluency where there was a weak positive correlation in both the *C9orf72* (r=0.15, *p* = 0.047) and *GRN* (r=0.15, *p* = 0.047) groups.

### Neural correlates of RSMS in each genetic group

**Fig. 3:**
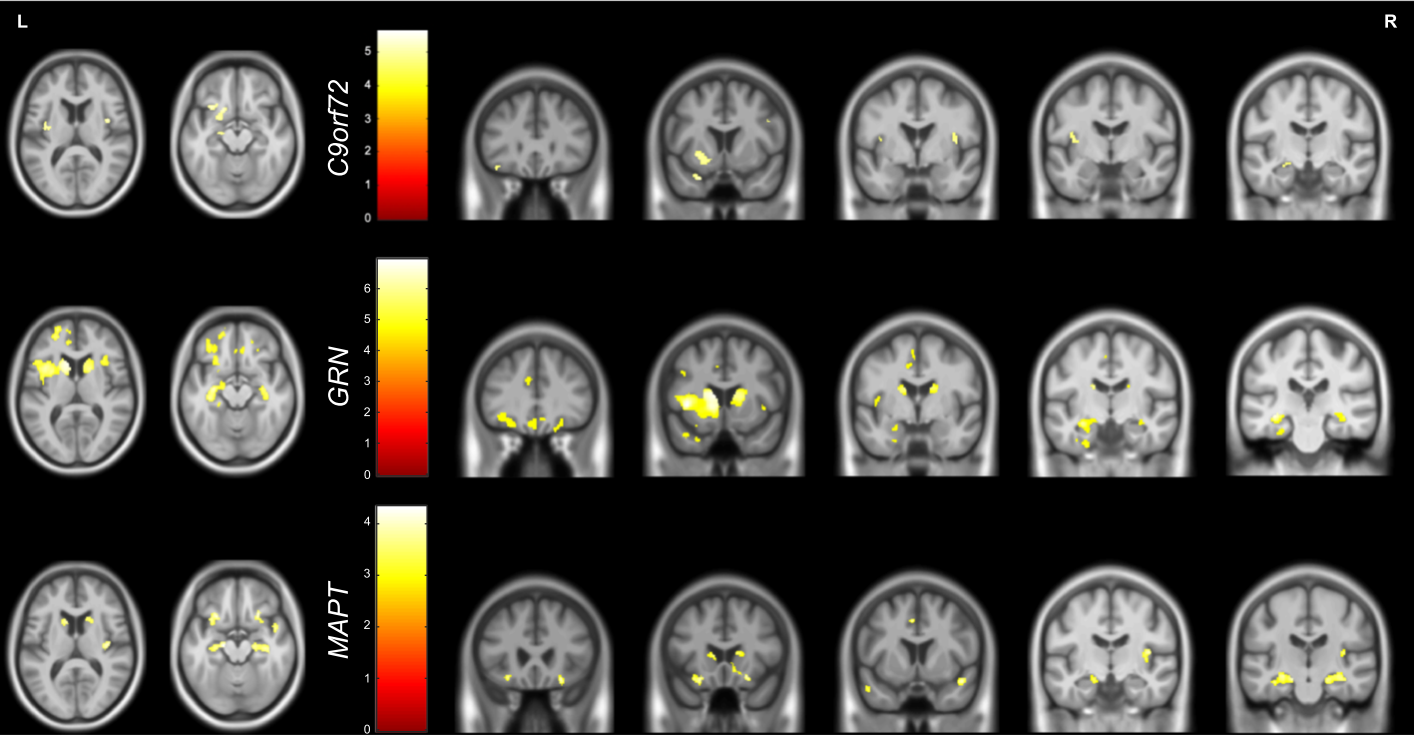
Neural correlates of RSMS total score. Results for *C9orf72* and *GRN* groups are shown corrected at *p* < 0.05, with results for the *MAPT* group shown at *p* < 0.001 uncorrected. Results are shown on a study-specific T1-weighted MRI template in MNI space

The VBM analysis revealed positive associations of the RSMS total score with grey matter volume corrected for multiple comparisons in the *C9orf72* and *GRN* groups, but only at an uncorrected p value of <0.001 for the *MAPT* group. Overlapping neural correlates were seen in each of the genetic groups, with an association of decreased score with lower grey matter volume in the orbitofrontal lobe, insula, temporal pole, medial temporal lobe and both caudate and putamen (Fig. 3, Table S6).

## Discussion

In this study, we have shown that the RSMS detects social cognitive impairment in genetic FTD, including early difficulties within the CDR 0.5 group of *C9orf72* mutation carriers for the total score and for both *C9orf72* and *GRN* mutation carriers for the modification of self-presentation (SP) subscore. RSMS total score is highly correlated with ‘CDR® plus NACC FTLD’ score and with an overlapping ‘social cognitive’ network of regions including orbitofrontal, anteromedial temporal, insula and striatal areas.

The results here show that the RSMS score decreases with increasing disease severity as measured by the CDR® plus NACC FTLD score, with a significant negative correlation between both scores in each genetic group i.e. RSMS decreases as CDR® plus NACC FTLD increases. This relationship has also been described in a recent study [14], although that study did not separate mutation carriers into separate genetic groups.

Carriers of *C9orf72* repeat expansions at CDR 0.5 (i.e. possibly or mildly symptomatic) perform significantly worse on the total RSMS score and both subscores than controls, whilst *GRN* mutation carriers have a significantly lower SP subscore and a trend to lower total and EX scores than controls. These results highlight the potential for the RSMS to detect early deficits in social cognition in these genetic cohorts of FTD, prior to phenoconversion to being fully symptomatic.

The profiles of RSMS performance in *MAPT* mutation carriers seem to be somewhat unique. Symptomatic *MAPT* mutation carriers scored much lower at baseline than the other *MAPT* mutation carriers, a result that is consistent with findings from other cross-sectional [5, 15–17] and longitudinal [18] familial FTD studies. This could imply that self-monitoring in *MAPT* mutation carriers is relatively stable in early disease stages until soon before or at the point of conversion when there is a rapid decline in social cognitive function, as opposed to a more gradual (and earlier) deterioration in *GRN* and *C9orf72* mutation carriers [19–22].

Analysis of the relationship of the RSMS with cognitive test scores reveals only very weak correlations, suggesting that the RSMS is likely to be measuring a different aspect of behaviour than the current psychometric tests. In *C9orf72* mutation carriers, there was a weak correlation with the Facial Emotion Recognition Test i.e. the ability to detect the emotions of others in their faces, suggesting some overlap in social cognitive abilities in performing these tasks within this group. However, the term social cognition encompasses a number of different skills which are dissociable [23], and socioemotional sensitivity (as measured by the RSMS) is likely to be represent a distinct (if nonetheless overlapping) domain. Weak correlations were also seen with category fluency in the *C9orf72* and *GRN* groups. The reason for this relationship is unclear but interestingly, prior studies have shown an association between verbal fluency and both social relationships and activity [24, 25], hypothesizing that fluency is better when social interaction can be maintained.

Previous studies in sporadic FTD have described links between deficits in empathic perspective taking and a ‘social cognition network’ comprising bifrontal (particularly orbitofrontal), anterior and inferior temporal and insula cortical regions [26–28]. Subcortical structures such as the amygdala and caudate have also been implicated in driving such dysfunction [28]. Results of the VBM analysis in this study highlighted frontal involvement across all mutation carrier groups, in particular the orbitofrontal cortex, a region known to be involved in decision-making and coordinating complex social and emotional behaviours [29–31] with its atrophy and circuitry disruption having been previously described in patients with behavioural variant FTD [32]. Previous studies specifically utilising the RSMS as a tool to measure social cognition have identified a positive association between socioemotional sensitivity and functional connectivity within the brain’s salience network, largely between the right anterior insula and both cortical and subcortical nodes [10], as well as between right supramarginal and angular gyri, and right frontal pole [33, 34]. Here, we demonstrate widespread insula involvement, anteriorly in *C9orf72* and *GRN* mutation carriers and posteriorly in *MAPT* mutation carriers, in addition to anterior cingulate cortex involvement in *GRN* mutation carriers exclusively, another crucial element of the salience network [35].

Other brain regions associated with such behavioural deficits in FTD include the inferior and medial temporal gyri [4], areas particularly involved in emotion perception and recognition. Grey matter volume of the temporal pole was positively correlated with RSMS score in all mutation groups, with *C9orf72* carriers also exhibiting an association with superior temporal gyrus and *GRN* and *MAPT* carriers showing a correlation with inferior temporal gyri specifically. Our results also show an association of the basal ganglia, particularly the caudate and putamen, in all genetic groups. These subcortical regions are also known to be implicated in emotion recognition [36–38], an integral factor in an individual’s performance on the RSMS.

Overall, there appears to be a network of brain regions associated with impairment of socioemotional sensitivity in FTD that includes frontal, temporal, insula and striatal areas, including significant crossover with areas involved in the salience network, thus supporting the established role of aberrant saliency detection in FTD-related social cognitive dysfunction.

### Limitations

These data should be interpreted in light of some limitations. Despite the large nature of GENFI in comparison to other FTD studies, one limitation lies in the relatively small numbers in some of the groups once stratified. Future studies should aim to replicate these findings in larger cohorts, as well as investigate longitudinal changes in socioemotional sensitivity over time.

Another limitation lies in the design of the RSMS, due to the inclusion of reverse scoring. While every effort is taken to ensure the informant understands how to answer correctly, we cannot eliminate the chance of misinterpretation.

Although the RSMS has been examined in a number of studies previously, and the data presented here suggests it could potentially be included as an outcome measure in genetic FTD trials, there has been limited validation of the questionnaire so far and more work will be necessary e.g. investigation of test-retest reliability.

Lastly, while global CDR® plus NACC FTLD scoring is a validated and robust tool used to measure disease severity in FTD, the assessment of motor and neuropsychiatric symptoms is not included. With FTD representing a diverse spectrum of symptomatic profiles, a limitation of this study lies in possible mis-categorisation of individuals who might be at a more advanced stage of their disease but present with symptoms that are not specifically addressed by this scale.

## Conclusions

In summary, this study describes the ability of the RSMS to detect early changes in socioemotional behaviour in distinct genetic cohorts of FTD and illustrates the neural correlates of self-monitoring in these populations. Whilst further studies will be needed to validate the RSMS and explore how it changes over time, the present data suggests it may well serve as a useful outcome measure in future clinical trials.

## Supplementary Information

**Additional file 1: Figure S1.** RSMS EX scores in each genetic carrier group, stratified by Global CDR^®^ plus NACC FTLD scores. Significant differences from controls and within each carrier group are starred. Differences between carrier groups are not shown. **Figure S2.** RSMS SP scores in each genetic carrier group, stratified by Global CDR^®^ plus NACC FTLD scores. Significant differences from controls and within each carrier group are starred. Differences between carrier groups are not shown. **Figure S3.** Negative correlations between RSMS total and CDR^®^ plus FTLD NACC SOB scores were observed across all mutation carrier groups: C9orf72 (r = -0.67, p < 0.001), GRN (r = -0.59, p < 0.001), MAPT (r = -0.53, p < 0.001). Each dot represents one mutation carrier. **Table S1.** RSMS total test scores (mean and SD) in healthy controls split by age group. **Table S2.** Cumulative frequency of RSMS total test scores in healthy controls. **Table S3.** Adjusted mean differences in RSMS EX scores between the genetic groups stratified by Global CDR^®^ plus NACC FTLD scores with 95% bias-corrected confidence intervals (significant values in bold). **Table S4.** Adjusted mean differences in RSMS SP scores between the genetic groups stratified by Global CDR^®^ plus NACC FTLD scores with 95% bias-corrected confidence intervals (significant values in bold). **Table S5.** Correlation of RSMS total test score with cognitive tests. Significant results are in bold. **Table S6.** Positive neuroanatomical correlates of grey matter volume with the RSMS total score in each genetic group.

## Data Availability

Data are available upon reasonable request. The raw data of this project are part of GENFI and are not publicly available in accordance with the ethical approval. Data can be accessed upon reasonable request to JDR (j.rohrer@ucl.ac.uk).

## Abbreviations

FTD: Frontotemporal dementia
RSMS: Revised Self-Monitoring Scale
GENFI: Genetic FTD initiative
*C9orf72*: Chromosome 9 open-reading frame 72
*GRN*: Progranulin
*MAPT*: Microtubule-associated protein tau
CDR® plus NACC FTLD: CDR® Dementia Staging Instrument with National Alzheimer Coordinating Centre Frontotemporal Lobar Degeneration component
VBM: Voxel-based morphometry
bvFTD: Behavioural variant FTD
CDR® plus NACC FTLD-SB: CDR® plus NACC FTLD sum of boxes
EX: RSMS socio-emotional expressiveness subscore
SP: RSMS modification of self-presentation subscore
SPM: Statistical parametric mapping
GM: Grey matter
WM: White matter
CSF: Cerebrospinal fluid
DARTEL: Fast-diffeomorphic image registration algorithm
MNI: Montreal Neurological Institute
TIV: Total intracranial volume
FWE: Family-Wise Error
