# Supplementary material for: The Revised Self-Monitoring Scale detects early impairment of social cognition in genetic frontotemporal dementia within the GENFI cohort
Source: Alzheimers Res Ther. 2021 Jul 12;13:127. doi: 10.1186/s13195-021-00865-w (PMC8276486; doi:10.1186/s13195-021-00865-w)
Supplement: Supplementary file 1 — Additional file 1: Figure S1. RSMS EX scores in each genetic carrier group, stratified by Global CDR® plus NACC FTLD scores. Significant differences from controls and within each carrier group are starred. Differences between carrier groups are not shown. Figure S2. RSMS SP scores in each genetic carrier group, stratified by Global CDR® plus NACC FTLD scores. Significant differences from controls and within each carrier group are starred. Differences between carrier groups are not shown. Figure S3. Negative correlations between RSMS total and CDR® plus FTLD NACC SOB scores were observed across all mutation carrier groups: C9orf72 (r = -0.67, p < 0.001), GRN (r = -0.59, p < 0.001), MAPT (r = -0.53, p < 0.001). Each dot represents one mutation carrier. Table S1. RSMS total test scores (mean and SD) in healthy controls split by age group. Table S2. Cumulative frequency of RSMS total test scores in healthy controls. Table S3. Adjusted mean differences in RSMS EX scores between the genetic groups stratified by Global CDR® plus NACC FTLD scores with 95% bias-corrected confidence intervals (significant values in bold). Table S4. Adjusted mean differences in RSMS SP scores between the genetic groups stratified by Global CDR® plus NACC FTLD scores with 95% bias-corrected confidence intervals (significant values in bold). Table S5. Correlation of RSMS total test score with cognitive tests. Significant results are in bold. Table S6. Positive neuroanatomical correlates of grey matter volume with the RSMS total score in each genetic group. [file 13195_2021_865_MOESM1_ESM.docx]

**Supplementary Data**

**Figure S1. RSMS EX scores in each genetic carrier group, stratified by Global CDR^®^ plus NACC FTLD scores. Significant differences from controls and within each carrier group are starred. Differences between carrier groups are not shown.**

**Figure S2. RSMS SP scores in each genetic carrier group, stratified by Global CDR^®^ plus NACC FTLD scores. Significant differences from controls and within each carrier group are starred. Differences between carrier groups are not shown.**

**Figure S3. Negative correlations between RSMS Total and CDR^®^ plus FTLD NACC SOB scores were observed across all mutation carrier groups: C9orf72 (r = -0.67, p < 0.001), GRN (r = -0.59, p < 0.001), MAPT (r = -0.53, p < 0.001). Each dot represents one mutation carrier.**


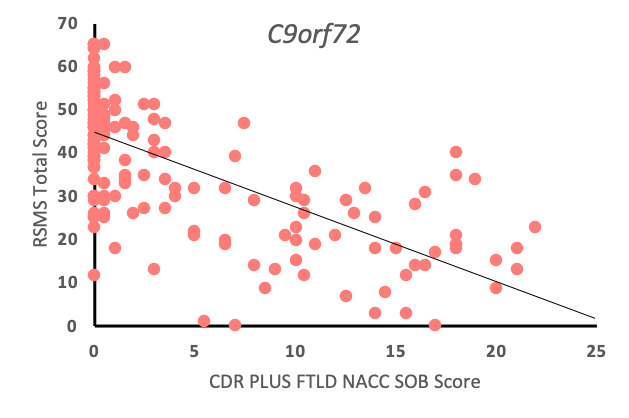

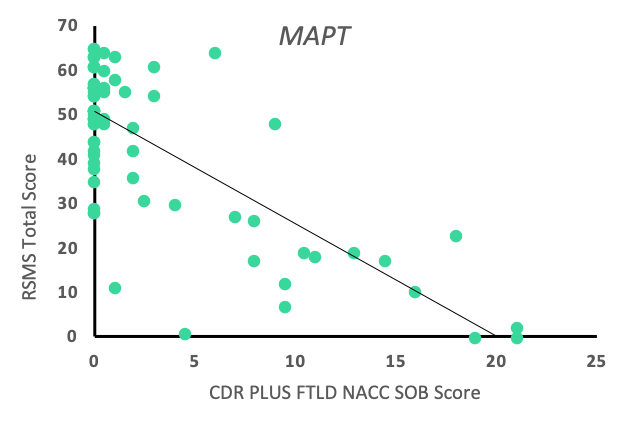

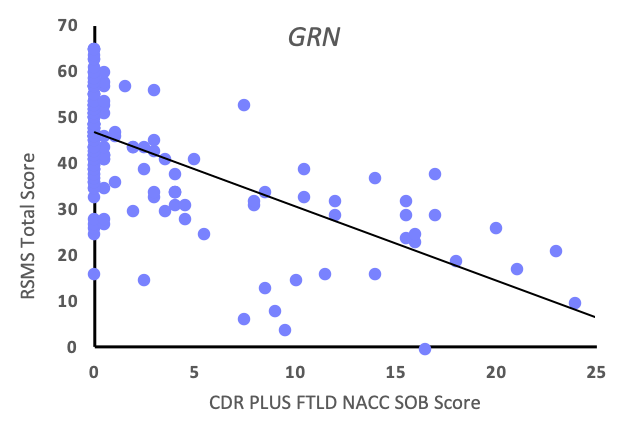


**Table S1: RSMS Total test scores (mean and SD) in healthy controls split by age group.**

| **Age group**  **(years)** | **All** | | | **Females** | | | **Males** | | |
| --- | --- | --- | --- | --- | --- | --- | --- | --- | --- |
|  | **N** | **Mean** | **SD** | **N** | **Mean** | **SD** | **N** | **Mean** | **SD** |
| **All** | 269 | 47.8 | 8.4 | 157 | 48.5 | 8.0 | 112 | 46.8 | 9.0 |
| **18.1-29.9** | 32 | 48.0 | 10.1 | 17 | 49.9 | 7.9 | 15 | 45.9 | 12.1 |
| **30.0-39.9** | 59 | 47.8 | 7.5 | 35 | 49.6 | 6.8 | 24 | 45.1 | 7.9 |
| **40.0-49.9** | 82 | 47.3 | 9.4 | 41 | 47.3 | 9.6 | 41 | 47.3 | 9.3 |
| **50.0-59.9** | 47 | 47.3 | 7.5 | 33 | 47.4 | 7.6 | 14 | 47.1 | 7.4 |
| **60.0-69.9** | 39 | 49.1 | 7.8 | 27 | 49.4 | 7.9 | 12 | 48.2 | 7.7 |
| **70.0+** | 10 | 49.3 | 7.7 | 4 | 50.0 | 2.7 | 6 | 48.8 | 10.0 |

**Table S2. Cumulative frequency of RSMS Total test scores in healthy controls.**

| **RSMS Total score** | **Number of participants** | **Cumulative frequency (%)** |
| --- | --- | --- |
| **17** | **1** | **0.4** |
| **21** | **1** | **0.7** |
| **26** | **1** | **1.1** |
| **27** | **1** | **1.5** |
| **28** | **1** | **1.9** |
| **30** | **2** | **2.6** |
| **31** | **1** | **3.0** |
| **32** | **3** | **4.1** |
| **33** | **5** | **6.0** |
| **34** | **1** | **6.3** |
| **35** | **1** | **6.7** |
| **36** | **4** | **8.2** |
| **37** | **6** | **10.4** |
| **38** | **4** | **11.9** |
| **39** | **9** | **15.2** |
| **40** | **11** | **19.3** |
| **41** | **7** | **21.9** |
| **42** | **8** | **24.9** |
| **43** | **14** | **30.1** |
| **44** | **7** | **32.7** |
| **45** | **8** | **35.7** |
| **46** | **17** | **42.0** |
| **47** | **9** | **45.4** |
| **48** | **17** | **51.7** |
| **49** | **16** | **57.6** |
| **50** | **14** | **62.8** |
| **51** | **12** | **67.3** |
| **52** | **9** | **70.6** |
| **53** | **12** | **75.1** |
| **54** | **10** | **78.8** |
| **55** | **12** | **83.3** |
| **56** | **4** | **84.8** |
| **57** | **3** | **85.9** |
| **58** | **11** | **90.0** |
| **59** | **6** | **92.2** |
| **60** | **4** | **93.7** |
| **61** | **4** | **95.2** |
| **62** | **1** | **95.5** |
| **63** | **4** | **97.0** |
| **64** | **2** | **97.8** |
| **65** | **6** | **100.0** |

**Table S3. Adjusted mean differences in RSMS EX scores between the genetic groups stratified by Global CDR^®^ plus NACC FTLD scores with 95% bias-corrected confidence intervals (significant values in bold).**

|  | | ***C9orf72*** | | | | | | ***GRN*** | | | | | | ***MAPT*** | | | | | | | |
| --- | --- | --- | --- | --- | --- | --- | --- | --- | --- | --- | --- | --- | --- | --- | --- | --- | --- | --- | --- | --- | --- |
|  |  | **0** | | **0.5** | | **1+** | | **0** | | **0.5** | | **1+** | | **0** | | **0.5** | | **1+** | | | |
| **Controls** | | -0.51 | | **-3.17** | | **-12.68** | | -0.01 | | -1.61 | | **-9.45** | | 0.36 | | 0.45 | | **-13.40** | | | |
|  |  | -1.75 | 0.72 | **-5.40** | **-0.93** | **-14.57** | **-10.78** | -0.81 | 0.80 | -4.20 | 0.98 | **-11.28** | **-7.62** | -1.18 | 1.90 | -3.74 | 4.65 | | **-17.46** | | **-9.33** |
| ***C9orf72*** | **0** |  | | **-2.65** | | **-12.16** | | 0.51 | | -1.09 | | **-8.94** | | 0.87 | | 0.97 | | **-12.88** | | | |
|  |  |  |  | **-5.23** | **-0.08** | **-14.47** | **-9.85** | -0.86 | 1.88 | -3.93 | 1.74 | **-11.16** | **-6.71** | -0.99 | 2.74 | -3.33 | 5.27 | **-17.10** | | **-8.67** | |
|  | **0.5** |  | |  | | **-9.51** | | **3.16** | | 1.56 | | **-6.28** | | **3.53** | | 3.62 | | **-10.23** | | | |
|  |  |  |  |  |  | **-12.21** | **-6.81** | **0.79** | **5.53** | -1.82 | 4.94 | **-9.03** | **-3.53** | **0.78** | **6.28** | -1.13 | 8.38 | **-14.73** | | **-5.72** | |
|  | **1+** |  | |  | |  | | **12.67** | | **11.07** | | **3.23** | | **13.04** | | **13.13** | | -0.72 | | | |
|  |  |  |  |  |  |  |  | **10.65** | **14.70** | **7.87** | **14.27** | **0.79** | **5.67** | **10.56** | **15.51** | **8.61** | **17.65** | -4.99 | | 3.55 | |
| ***GRN*** | **0** |  | |  | |  | |  | | -1.60 | | **-9.44** | | 0.37 | | 0.46 | | **-13.39** | | | |
|  |  |  |  |  |  |  |  |  |  | -4.34 | 1.14 | **-11.37** | **-7.51** | -1.34 | 2.07 | -3.82 | 4.74 | **-17.49** | | **-9.29** | |
|  | **0.5** |  | |  | |  | |  | |  | | **-7.84** | | 1.97 | | 2.06 | | **-11.79** | | | |
|  |  |  |  |  |  |  |  |  |  |  |  | **-10.83** | **-4.85** | -0.90 | 4.84 | -2.75 | 6.87 | **-16.47** | | **-7.11** | |
|  | **1+** |  | |  | |  | |  | |  | |  | | **9.81** | | **9.90** | | -3.95 | | | |
|  |  |  |  |  |  |  |  |  |  |  |  |  |  | **7.37** | **12.25** | **5.36** | **14.44** | -8.29 | | 0.39 | |
| ***MAPT*** | **0** |  | |  | |  | |  | |  | |  | |  | | 0.09 | | **-13.76** | | | |
|  |  |  |  |  |  |  |  |  |  |  |  |  |  |  |  | -3.96 | 4.15 | **-18.08** | | **-9.44** | |
|  | **0.5** |  | |  | |  | |  | |  | |  | |  | |  | | **-13.85** | | | |
|  |  |  |  |  |  |  |  |  |  |  |  |  |  |  |  |  |  | **-19.42** | | **-8.29** | |
|  | **1+** |  | |  | |  | |  | |  | |  | |  | |  | |  | | | |

**Table S4. Adjusted mean differences in RSMS SP scores between the genetic groups stratified by Global CDR^®^ plus NACC FTLD scores with 95% bias-corrected confidence intervals (significant values in bold).**

|  | | ***C9orf72*** | | | | | | ***GRN*** | | | | | | ***MAPT*** | | | | | |
| --- | --- | --- | --- | --- | --- | --- | --- | --- | --- | --- | --- | --- | --- | --- | --- | --- | --- | --- | --- |
|  |  | **0** | | **0.5** | | **1+** | | **0** | | **0.5** | | **1+** | | **0** | | **0.5** | | **1+** | |
| **Controls** | | -0.40 | | **-2.21** | | **-9.74** | | -0.35 | | **-2.44** | | **-7.98** | | 1.66 | | 1.58 | | **-10.87** | |
|  |  | -1.86 | 1.06 | **-4.39** | **-0.03** | **-11.62** | **-7.86** | -1.61 | 0.92 | **-4.84** | **-0.04** | **-9.91** | **-6.06** | -0.32 | 3.64 | -2.29 | 5.45 | **-15.15** | **-6.60** |
| ***C9orf72*** | **0** |  | | -1.81 | | **-9.34** | | 0.05 | | -2.04 | | **-7.58** | | 2.06 | | 1.98 | | **-10.47** | |
|  |  |  |  | -4.32 | 0.70 | **-11.65** | **-7.03** | -1.71 | 1.82 | -4.78 | 0.69 | **-9.96** | **-5.21** | -0.24 | 4.36 | -2.05 | 6.01 | **-14.96** | **-5.99** |
|  | **0.5** |  | |  | | **-7.53** | | 1.86 | | -0.23 | | **-5.77** | | **3.87** | | 3.79 | | **-8.66** | |
|  |  |  |  |  |  | **-10.24** | **-4.83** | -0.58 | 4.31 | -3.33 | 2.87 | **-8.51** | **-3.04** | **0.99** | **6.75** | -0.48 | 8.06 | **-13.36** | **-3.96** |
|  | **1+** |  | |  | |  | | **9.40** | | **7.30** | | 1.76 | | **11.40** | | **11.32** | | -1.13 | |
|  |  |  |  |  |  |  |  | **7.31** | **11.48** | **4.50** | **10.10** | -0.77 | 4.28 | **8.78** | **14.03** | **7.27** | **15.37** | -5.61 | 3.35 |
| ***GRN*** | **0** |  | |  | |  | |  | | -2.10 | | **-7.64** | | 2.01 | | 1.92 | | **-10.53** | |
|  |  |  |  |  |  |  |  |  |  | -4.75 | 0.56 | **-9.71** | **-5.56** | -0.29 | 4.31 | -2.12 | 5.97 | **-14.94** | **-6.12** |
|  | **0.5** |  | |  | |  | |  | |  | | **-5.54** | | **4.10** | | 4.02 | | **-8.43** | |
|  |  |  |  |  |  |  |  |  |  |  |  | **-8.53** | **-2.55** | **1.05** | **7.16** | -0.35 | 8.40 | **-13.08** | **-3.78** |
|  | **1+** |  | |  | |  | |  | |  | |  | | **9.64** | | **9.56** | | -2.89 | |
|  |  |  |  |  |  |  |  |  |  |  |  |  |  | **6.88** | **12.41** | **5.40** | **13.72** | -7.47 | 1.69 |
| ***MAPT*** | **0** |  | |  | |  | |  | |  | |  | |  | | -0.08 | | **-12.53** | |
|  |  |  |  |  |  |  |  |  |  |  |  |  |  |  |  | -3.93 | 3.77 | **-17.24** | **-7.82** |
|  | **0.5** |  | |  | |  | |  | |  | |  | |  | |  | | **-12.45** | |
|  |  |  |  |  |  |  |  |  |  |  |  |  |  |  |  |  |  | **-17.85** | **-7.05** |
|  | **1+** |  | |  | |  | |  | |  | |  | |  | |  | |  | |

**Table S5. Correlation of RSMS Total test score with cognitive tests. Significant results are in bold.**

| **Cognitive test** | **Genetic group** | **rho** | **p-value** |
| --- | --- | --- | --- |
| **Trail Making Test Part A** | ***C9orf72*** | -0.08 | 0.289 |
|  | ***GRN*** | -0.08 | 0.275 |
|  | ***MAPT*** | 0.01 | 0.930 |
| **Trail Making Test Part B** | ***C9orf72*** | -0.04 | 0.627 |
|  | ***GRN*** | -0.08 | 0.278 |
|  | ***MAPT*** | 0.02 | 0.902 |
| **Digit Symbol test** | ***C9orf72*** | 0.11 | 0.165 |
|  | ***GRN*** | 0.02 | 0.746 |
|  | ***MAPT*** | 0.12 | 0.335 |
| **D-KEFS Color-Word Interference Test Ink Naming** | ***C9orf72*** | -0.10 | 0.183 |
|  | ***GRN*** | 0.01 | 0.923 |
|  | ***MAPT*** | -0.21 | 0.090 |
| **Category fluency** | ***C9orf72*** | **0.15** | **0.047** |
|  | ***GRN*** | **0.17** | **0.027** |
|  | ***MAPT*** | 0.19 | 0.123 |
| **Faux Pas recognition test** | ***C9orf72*** | 0.06 | 0.431 |
|  | ***GRN*** | -0.03 | 0.690 |
|  | ***MAPT*** | 0.04 | 0.737 |
| **Facial Emotion Recognition test** | ***C9orf72*** | **0.18** | **0.018** |
|  | ***GRN*** | 0.04 | 0.649 |
|  | ***MAPT*** | -0.01 | 0.916 |

**Table S6. Positive neuroanatomical correlates of grey matter volume with the RSMS Total score in each genetic group.**

| **Genetic group** | **Neuroanatomical region** | **Cluster** | **T** | **Peak** | | **Coordinates (mm)** | | |
| --- | --- | --- | --- | --- | --- | --- | --- | --- |
|  |  |  |  | **p(FWE-corrected)** | **p(uncorrected)** | **x** | **y** | **z** |
| ***C9orf72*** | Left putamen, anterior insula and orbitofrontal gyrus | 534 | 5.73 | 0.001 | < 0.001 | -16 | 18 | -12 |
|  | Left superior temporal sulcus | 83 | 5.57 | 0.002 | < 0.001 | -46 | -42 | 16 |
|  | Left superior temporal gyrus | 21 | 5.52 | 0.003 | < 0.001 | -48 | -27 | 2 |
|  | Right insula | 56 | 5.40 | 0.005 | < 0.001 | 39 | 0 | 9 |
|  | Left caudate | 26 | 5.40 | 0.005 | < 0.001 | -6 | 4 | -3 |
|  | Right inferior frontal gyrus | 51 | 5.32 | 0.007 | < 0.001 | 48 | 6 | 28 |
|  | Left temporal pole | 48 | 5.30 | 0.007 | < 0.001 | -27 | 8 | -28 |
|  | Left anterior insula | 85 | 5.28 | 0.008 | < 0.001 | -32 | 18 | -14 |
|  | Right orbitofrontal gyrus | 26 | 5.10 | 0.017 | < 0.001 | 28 | 15 | -21 |
|  | Left putamen | 92 | 5.08 | 0.018 | < 0.001 | -34 | -8 | 9 |
|  | Left hippocampus | 17 | 4.95 | 0.030 | < 0.001 | -24 | -12 | -15 |
| ***GRN*** | Left anterior insula, caudate and putamen | 5604 | 6.91 | < 0.001 | < 0.001 | -38 | 12 | 3 |
|  | Left hippocampus and amygdala | 1712 | 6.73 | < 0.001 | < 0.001 | -34 | -26 | -9 |
|  | Right caudate | 1108 | 6.15 | < 0.001 | < 0.001 | 14 | 4 | 18 |
|  | Right hippocampus | 324 | 6.06 | < 0.001 | < 0.001 | 33 | -16 | -10 |
|  | Left inferior frontal gyrus | 470 | 5.93 | < 0.001 | < 0.001 | -39 | 6 | 26 |
|  | Left middle frontal gyrus | 367 | 5.76 | 0.001 | < 0.001 | -26 | 50 | 4 |
|  | Right superior parietal lobule | 36 | 5.59 | 0.002 | < 0.001 | 30 | -40 | 46 |
|  | Left middle cingulate gyrus | 75 | 5.54 | 0.002 | < 0.001 | -9 | -4 | 42 |
|  | Left middle frontal gyrus | 204 | 5.51 | 0.003 | < 0.001 | -34 | 20 | 52 |
|  | Left anterior cingulate gyrus | 457 | 5.51 | 0.003 | < 0.001 | -9 | 38 | 22 |
|  | Right anterior insula | 593 | 5.46 | 0.003 | < 0.001 | 34 | 22 | 4 |
|  | Left frontal pole | 110 | 5.44 | 0.004 | < 0.001 | -16 | 62 | -9 |
|  | Right orbitofrontal gyrus | 90 | 5.36 | 0.005 | < 0.001 | 20 | 24 | -24 |
|  | Right middle frontal gyrus | 42 | 5.34 | 0.005 | < 0.001 | 32 | 20 | 50 |
|  | Left superior frontal gyrus | 57 | 5.33 | 0.006 | < 0.001 | -12 | 56 | 20 |
|  | Left superior frontal gyrus | 101 | 5.28 | 0.007 | < 0.001 | -10 | 57 | 4 |
|  | Right middle frontal gyrus | 34 | 5.26 | 0.007 | < 0.001 | 24 | 50 | 12 |
|  | Left precentral gyrus | 28 | 5.24 | 0.008 | < 0.001 | -54 | -6 | 48 |
|  | Left entorhinal cortex | 139 | 5.23 | 0.008 | < 0.001 | -26 | 3 | -33 |
|  | Left orbitofrontal cortex | 221 | 5.23 | 0.008 | < 0.001 | -3 | 30 | -20 |
|  | Right orbitofrontal gyrus | 124 | 5.21 | 0.009 | < 0.001 | 20 | 34 | -24 |
|  | Left inferior temporal gyrus | 37 | 5.19 | 0.010 | < 0.001 | -36 | -4 | -36 |
|  | Left temporal pole | 93 | 5.18 | 0.010 | < 0.001 | -40 | 12 | -28 |
|  | Left precentral gyrus | 24 | 5.15 | 0.012 | < 0.001 | -18 | -15 | 70 |
|  | Left temporal pole | 44 | 5.14 | 0.012 | < 0.001 | -32 | 3 | -44 |
|  | Left supplementary motor cortex | 99 | 5.12 | 0.013 | < 0.001 | -6 | 2 | 58 |
|  | Right orbitofrontal gyrus | 64 | 5.11 | 0.013 | < 0.001 | 16 | 46 | -15 |
|  | Right anterior cingulate gyrus | 46 | 5.11 | 0.014 | < 0.001 | 8 | 36 | -12 |
|  | Left cingulate gyrus | 28 | 5.02 | 0.019 | < 0.001 | -9 | -52 | 3 |
| ***MAPT*** | Left hippocampus | 393 | 4.32 | 0.496 | < 0.001 | -16 | -16 | -16 |
|  | Left temporal pole | 117 | 4.28 | 0.534 | < 0.001 | -52 | 12 | -28 |
|  | Left ventromedial prefrontal cortex | 90 | 4.23 | 0.589 | < 0.001 | -4 | 4 | 45 |
|  | Right orbitofrontal gyrus | 129 | 4.20 | 0.612 | < 0.001 | 24 | 21 | -15 |
|  | Left orbitofrontal gyrus | 281 | 4.13 | 0.69 | < 0.001 | -27 | 21 | -16 |
|  | Right posterior insula | 165 | 4.10 | 0.712 | < 0.001 | 39 | -14 | 12 |
|  | Right superior parietal lobe | 58 | 3.96 | 0.837 | < 0.001 | 34 | -57 | 51 |
|  | Right temporal pole | 148 | 3.92 | 0.866 | < 0.001 | 46 | 6 | -18 |
|  | Right hippocampus | 388 | 3.88 | 0.895 | < 0.001 | 33 | -20 | -15 |
|  | Left caudate | 188 | 3.87 | 0.899 | < 0.001 | -10 | 15 | 10 |
|  | Left superior frontal gyrus | 74 | 3.82 | 0.925 | < 0.001 | -10 | 60 | 21 |
|  | Left orbitofrontal gyrus | 63 | 3.65 | 0.981 | < 0.001 | -14 | 30 | -24 |
|  | Right caudate | 189 | 3.61 | 0.987 | < 0.001 | 14 | 21 | 8 |
|  | Left inferior temporal gyrus | 59 | 3.55 | 0.994 | < 0.001 | -42 | 2 | -45 |
|  | Left putamen | 63 | 3.50 | 0.997 | < 0.001 | -15 | 12 | -9 |
